# The role of soluble tumor necrosis factor like weak inducer of apoptosis and interleukin-17A in the etiopathogenesis of celiac disease: A cross-sectional study

**DOI:** 10.1097/MD.0000000000003937

**Published:** 2016-07-01

**Authors:** Mahmut Yuksel, Mustafa Kaplan, Ihsan Ates, Zeki Mesut Yalın Kilic, Hasan Kilic, Nuretdin Suna, Hale Ates, Ertugrul Kayacetin

**Affiliations:** a Turkey Yuksek Ihtisas Training and Research Hospital, Department of Gastroenterology; b Ankara Numune Training and Research Hospital, Department of Internal Medicine; c Turkey Yuksek Ihtisas Training and Research Hospital, Department of Microbiology; d Atatürk Chest Diseases & Thoracic Surgery Training and Research Hospital, Department of Immunology and Allergy, Ankara, Turkey.

**Keywords:** antigliadin, gluten, T helper 1, T helper 17, tissue transglutaminase, TNF superfamily

## Abstract

Our aim in this study was to determine soluble tumor necrosis factor (TNF)-like weak inducer of apoptosis (sTWEAK) and interleukin-17A (IL-17A) levels in celiac disease, and their association with the gluten diet and autoantibodies. Eighty patients with celiac diagnosis and 80 healthy control individuals with similar age, gender and body mass index to the patient group were included in the study. Serum sTWEAK and IL-17A levels were measured by the serum enzyme-linked immunosorbent assay kit. The median IL-17A (117.5 pg/mL vs. 56.7 pg/mL; *P* = 0.001) level in celiac patients was higher than in the control group, while the median sTWEAK (543 pg/mL vs. 643 pg/mL; *P* = 0.016) level in patients was determined to be lower. In the patient group, patients who complied with the gluten diet had a lower level of median IL-17A (98.1 pg/mL vs. 197.5 pg/mL; *P* = 0.034) and a higher level of sTWEAK (606 pg/mL vs. 522.8 pg/mL; *P* = 0.031) than those who did not adhere. Furthermore, the IL-17A level was higher and the sTWEAK level was lower in celiac patients with positive antibody than those with negative antibody. A positive correlation was determined among anti-gliadin antibody IgA, anti-gliadin antibody IgG, anti-tissue transglutaminase IgG levels and the IL-17A level, and a negative correlation was determined with the sTWEAK level. In celiac disease, the sTWEAK and IL-17A levels differ between patients who cannot adapt to the gluten diet and who are autoantibody positive, and patients who adapt to the diet and are autoantibody negative. We believe that sTWEAK and IL-17A are associated with the inflammation in celiac pathogenesis.

## 1 Introduction

Celiac disease is an autoimmune disease that occurs in genetically susceptible individuals and progresses by intestinal inflammation.^[1,2]^ It is thought that major histocompatibility complex class II (MHC II) DQ2 and DQ8 genes are associated with this disease.^[3]^ After gluten intake in genetically susceptible patients, humoral and cellular immunity is triggered. A chronic inflammation occurs in the whole body, primarily in the intestinal mucosa.^[4]^

With gluten intake, gluten peptides bound to human leukocyte antigens and are recognized by T cells. T cells initiate the inflammatory process via T helper1 (Th1),^[5]^ T helper 2 (Th2), and T helper 17 (Th17).^[6]^ With the activation of T cells, the release of cytokines and the immunogenicity and activation of disease-specific antigens increase.^[7]^ With the activation of these antigens, B cells start reproducing clonally and antibodies are produced. The anti-gliadin antibody (A is formed against gliadin, the protein part of gluten, and the tissue transglutaminase (t TG) antibody (Anti-t TG) is formed against the t TG enzyme, which can be found in many tissues within the body.^[1,8]^ Autoantibodies increasing secondarily to the inflammatory response causes tissue damage in the gastrointestinal system and therefore causes perfusion failure, therefore, ischemia, necrosis, and cell lysis. Damage in the cell and oxidative stress as a result of the tissue ischemia increase the inflammation level more. Therefore, the tissue damage and chronic inflammation level in the celiac patients are expected to be higher in celiac patients than in patients in remission. In other words, increase of autoantibodies in celiac disease is the result of inflammation and the initiator of the inflammation.

With the activation of T cells, Th1 and Th17 are synthesized, and, therefore, the level of proinflammatory cytokines increases. Tumor necrosis factor (TNF) subgroups are expected to increase via Th1 and interleukin 17 (IL 17) levels are expected to increase via Th17.

A TNF-like weak inducer of apoptosis (TWEAK), which is a cytokine from the TNF superfamily, shows its activity via the fibroblast growth factor-inducible 14 (Fn14) receptor.^[9]^ TWEAK has two active forms as soluble and membrane bound.^[10]^ Soluble TWEAK (sTWEAK) plays an important role in inflammation, apoptosis, and atherosclerosis.^[11]^

While IL 17 cytokine is synthesized by macrophages, natural killer T cells, dendritic cells, microglia, and neutrophils, it is also primarily synthesized by Th17.^[12]^ IL 17 has a total of 6 different forms (IL-17 A-B-C-D-E-F), including interleukin-17A (IL-17A), which play a critical role in the inflammatory process. IL-17A is a proinflammatory cytokine that plays a major role in the activation and migration of neutrophils.^[13]^

Recent studies have frequently emphasized the role of sTWEAK and IL-17A in inflammatory and autoimmune diseases.^[14,15]^ While there are a limited number of studies on IL-17A in celiac patients,^[3,16]^ we have not come across a study that examines the sTWEAK level in this disease.

Therefore, in this study, we aimed to determine the sTWEAK and IL-17A levels in celiac patents and their association with the gluten diet and autoantibodies.

## 2 Methods

### 2.1 Study population

This study was conducted between April and August 2015 at the Turkey Yuksek Ihtisas Training and Research Hospital's Gastroenterology Clinic and the Ankara Numune Training and Research Hospital's Internal Medicine Clinic.

A total of 160 participants, 80 of whom were patients with celiac diagnosis and 80 of whom are healthy volunteers with no known diseases were included in this study.

The celiac group was made up of active patients and patients in remission who are under clinical supervision, over the age of 18, and with or without adherence to the diet. The patients who were non-compliant to a gluten-free diet and had clinical symptoms of stomachache, distension, diarrhea, and nausea, and the patients with high levels of C-reactive protein (CRP) at the laboratory were considered as the active celiac patients. The patients who had no symptoms, were compliant to the gluten-free diet, and had CRP levels within the normal limits were considered as celiac patients in remission. The patient group, which had a diet with no gluten for 3 months was considered as the patient group compliant to the gluten-free diet. Patients’ compliance to the diet was confirmed with clinical and laboratory findings.

The healthy control group was formed of healthy volunteers who applied to our hospital for a check-up and who had no known diseases or history of drug use. The healthy control group was included in the study based on their similarities to the patient group in age, gender, and body mass index (BMI) and in their order of application.

Patients who had known chronic illnesses, rheumatism or infective inflammatory diseases, acute liver or renal failure, thyroid dysfunction, or immunosuppressive drug use, and who were unsupervised were not included in the study.

The study was designed in accordance with the 2013 Brazil version of the Declaration of Helsinki. It was approved by the local Ethical Board Research Commission (date: June 18, 2014, protocol number: 320). Written consent was taken from all participants who were included in the study.

### 2.2 Biochemical parameters

Venous blood samples were taken from participants between the hours of 8 and 10 AM after 8 hours of fasting in order to measure laboratory parameters. The blood samples were then centrifuged for 10 minutes at 4000 rpm and separated. The serum samples were kept at −80 °C until all samples were collected. Later, laboratory parameters in all samples were examined during the same session.

The laboratory parameters, anti gliadin antibodies IgA (AGA-IgA), anti gliadin antibodies IgG (AGA-IgG), anti-tissue Transglutaminase IgA antibodies (Anti-t TGA), and anti-tissue Transglutaminase IgA antibodies (Anti-t TGG) were measured by electroilluminescence immunoassay method using Cobas e 601 (Roche Diagnostics Corp., Indianapolis, IN) analyzer. CRP was measured by immunoturbidimetric method using Hitachi Modular P800 (Roche Diagnostics Corp., Indianapolis, IN) analyzer. The erythrocyte and platelet count, leukocyte count, and other hemogram parameters were measured via the impedance (resistance) method using a Sysmex XE 2100 optic laser scatter hematology analyzer (Roche Diagnostic, Corp., Indianapolis, IN), and hemoglobin was quantified photometrically.

### 2.3 sTWEAK measurement

Soluble TNFs like weak inducer of apoptosis levels were measured by the serum enzyme-linked immunosorbent assay (ELISA) kit (eBioscience, Human TWEAK Instant ELISA, Cat no: BMS2006INST). The calculated overall intra-assay and inter-assay coefficient of variation was 7.9% to 9.2%. The results were expressed in pg/mL.

### 2.4 IL-17A measurement

Interleukin-17 levels were measured by the serum ELISA kit (BOSTER IMMUNOLEADER, human IL 17, Cat no: EK0430). The results were expressed in pg/mL.

### 2.5 Statistical analysis

The Statistical Package for Social Sciences (SPSS) for Windows 20 (IBM SPSS Inc., Chicago, MI) software was used for statistical assessments. The Kolmogorov–Smirnov test was utilized to determine the distribution of data. Continuous variables with normal distribution were expressed as mean ± standard deviation, and continuous variables without normal distribution were expressed as median [interquartile range]. Categorical variables were presented as numbers and percentage. Continuous variables with normal distribution were compared with an independent sample *t*-test where appropriate. Continuous variables with non-normal distribution were compared with the Mann–Whitney *U* test where appropriate. The relationship between the numeric parameters was analyzed by Pearson and Spearman correlation analysis. Other risk factors were adjusted with partial correlation and the association between AGA and anti-t TG antibodies, and sTWEAK and IL-17A levels were examined. A *P* value <0.05 was considered significant for statistical analyses.

## 3 Results

The demographic, characteristics, and laboratory findings of study population have been summarized in Table 1. The study population was composed of 80 (24 men, 56 women, mean age: 44.6 ± 13.4 years) celiac patients and 80 (15 men, 65 women, mean age: 44.2 ± 14 years) healthy control group volunteers. A significant difference in terms of age, gender, and BMI levels were not determined in either group (*P* > 0.05). Smoking and alcohol use in both groups were similar (*P* > 0.05). In supervised celiac patients, the median duration of the disease was determined to be 6 years.

**Table 1:**
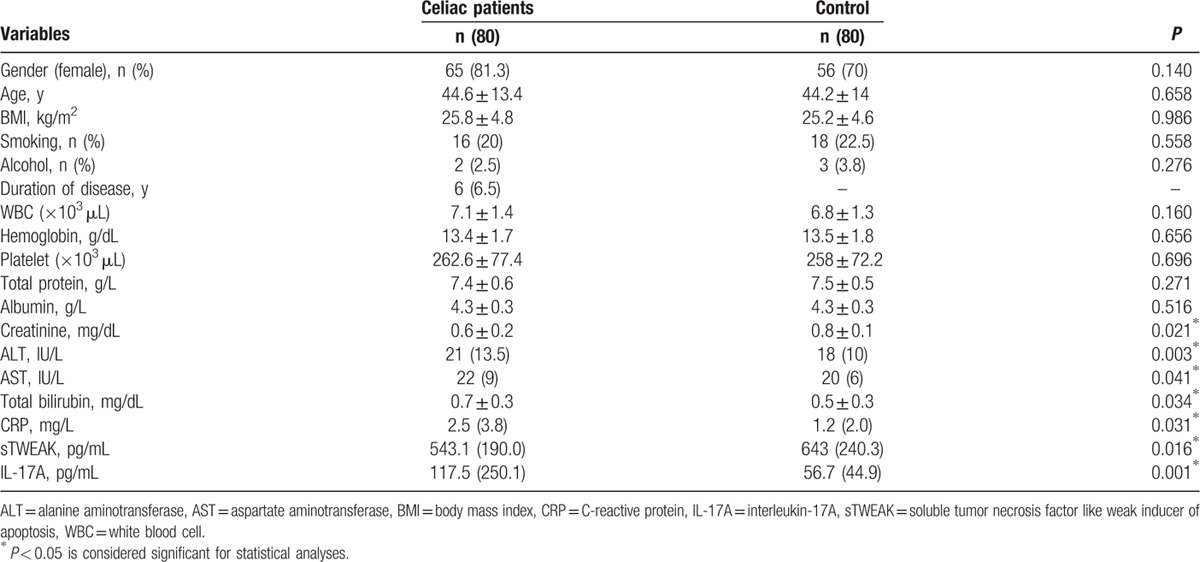
The demographic characteristics and laboratory findings of the study population.

The mean creatinine level (0.6 ± 0.1 mg/dL vs. 0.8 ± 0.1 mg/dL, respectively; *P* = 0.021) was determined to be lower in celiac patients than the control group. The median alanine aminotransferase (21 IU/L vs. 18 IU/L, respectively; *P* = 0.003), aspartate aminotransferase (22 IU/L vs. 20 IU/L, respectively; *P* = 0.041), and CRP (2.5 mg/L vs. 1.2 mg/L, respectively; *P* = 0.031) levels and the mean total bilirubin (0.7 ± 0.3 mg/dL vs. 0.5 ± 0.3 mg/dL, respectively; *P* = 0.034) level were determined to be higher in celiac patients than the control group. A significant difference was not determined in terms of other biochemical and hemogram parameters (*P* > 0.05).

**Figure 1:**
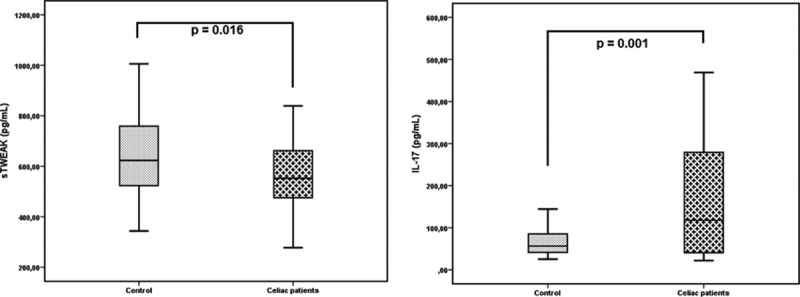
The distribution of sTWEAK and IL-17A levels between celiac patients and the control group. IL-17A = interleukin-17A, sTWEAK = soluble tumor necrosis factor like weak inducer of apoptosis.

In celiac patients, the median IL-17A (117.5 pg/mL vs. 56.7 pg/mL, respectively; *P* = 0.001) was determined to be higher, and the median sTWEAK (543 pg/mL vs. 643 pg/mL, respectively; *P* = 0.016) level was determined to be lower, when compared with the control group (Fig. 1).

In the celiac group, patients with adherence to the diet had a lower median IL-17A (98.1 pg/mL vs. 197.5 pg/mL, respectively; *P* = 0.034) level and a higher median sTWEAK (606 pg/mL vs. 522.8 pg/mL; *P* = 0.031) level than patients without adherence to the diet. IL-17A and sTWEAK levels based on the positivity or negativity of AGA (IgA and IgG) and anti-t TG (IgA and IgG) antibodies have been shown in detail in Table 2.

**Table 2:**
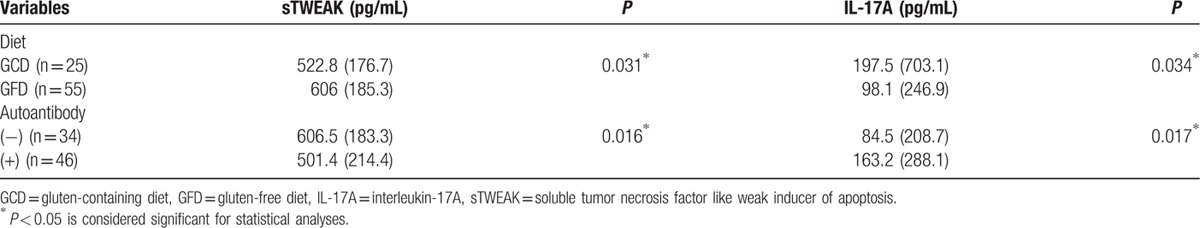
sTWEAK and IL-17A levels based on diet compliance and antibody positivity in celiac group.

In the patient group, the association between sTWEAK and IL-17A with celiac antibodies and other parameters has been shown in detail in Table 3. A negative correlation was determined between the sTWEAK level and IL-17A (*r* = −0.567, *P* = 0.009) and CRP (*r* = −0.280, *P* = 0.012). A positive correlation was determined between IL-17A and CRP (*r* = 0.302, *P* = 0.013). A positive correlation between AGA-IgA and AGA-IgG levels and the IL-17A level, and a negative correlation with the sTWEAK level was determined. A negative correlation was determined between the anti-t TGA level and the sTWEAK (*r* = −0.282, *P* = 0.011) level. A positive correlation was determined between the anti-t TGG level and the IL-17A (*r* = 0.326, *P* = 0.003) level. The association between the sTWEAK and IL-17A levels and other parameters has been shown in detail in Table 3.

**Table 3:**
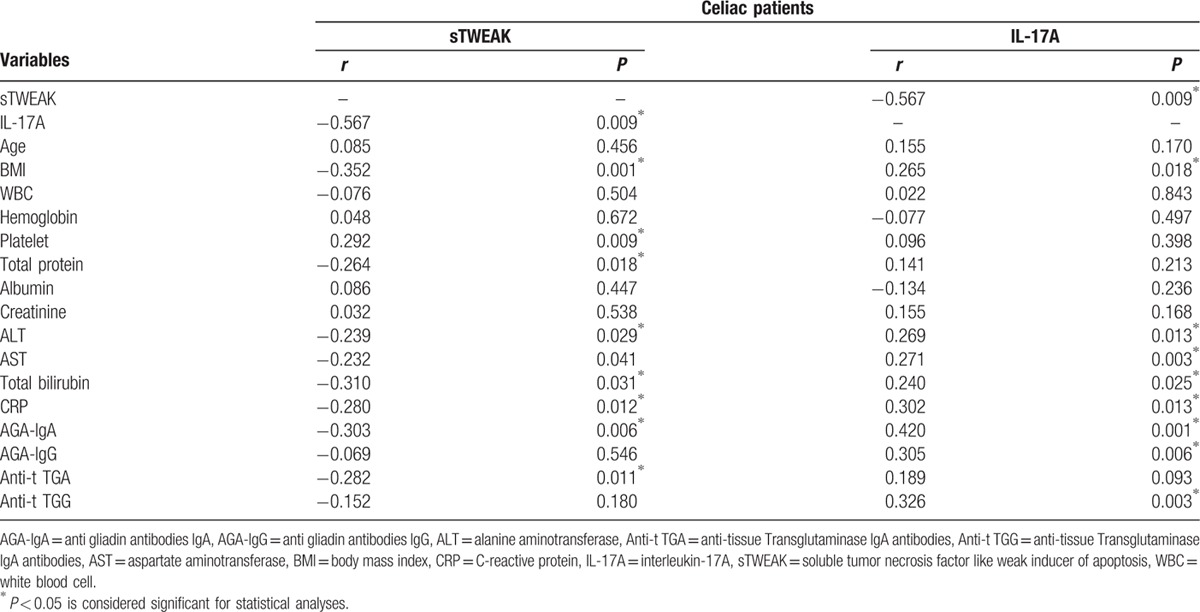
The association of sTWEAK and IL-17A with other parameters in celiac patients.

During the correlation analysis, when risk factors, other than celiac antibodies, associated with sTWEAK and IL-17A levels were cleared, it was determined that the association between the AGA (IgA and IgG) and anti-t TG (IgA and IgG) antibody levels and the sTWEAK and IL-17A levels continued (Table 4).

**Table 4:**
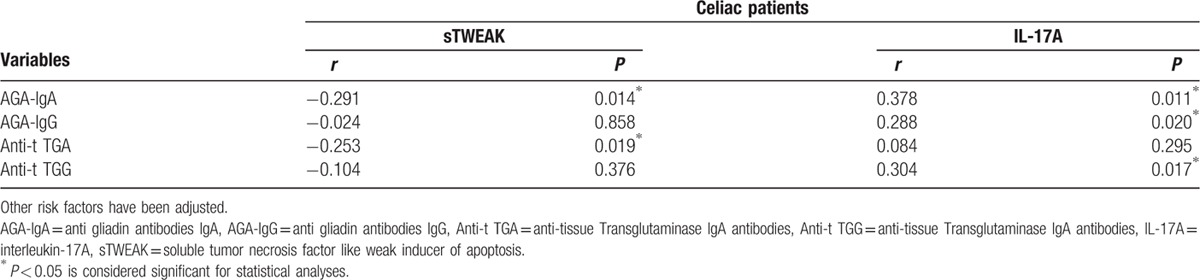
The association between sTWEAK and IL-17A and antibody levels in celiac patients when other risk factors are adjusted.

## 4 Discussion

Our study has determined a lower sTWEAK level and a higher IL-17A level in celiac patients than in the control group. Regarding celiac patients, those with autoantibody-positive rather than autoantibody-negative and also patients following the gluten-free diet rather than those not adhering with the diet, had lower levels of sTWEAK and higher levels of IL-17A. The correlation analysis has shown that celiac antibodies are associated with these proinflammatory cytokines. As far as we are aware, this is the first study that examines the sTWEAK level in celiac patients.

Innate and acquired T-cell-mediated immune system plays an important role in celiac disease.^[17]^ Deamidated gluten peptides are presented to CD4+ Th cells with subsequent release of proinflammatory cytokines. CD4+ Th1 lymphocytes in the lamina propria mediate T-cell mediated immune response. Another proinflammatory cytokine associated with CD4+ T cells found in the lamina propria and is thought to play a role in celiac pathogenesis is IL-17A.^[18]^ IL-17A causes the granulocyte colony stimulant factor (G-CSF) secretion to increase by stimulating epithelium cells. With the increase of G-CSF, neutrophil granulopoiesis is induced, therefore causing the increase of neutrophils and activation.^[19]^ As a result, an excessive inflammatory response occurs. After these explanations, we can say that after gluten intake in celiac disease a T-cell-mediated immune response occurs and proinflammatory cells such as Th1 and Th17 play a role in this immune response.^[20]^ While Th17 shows its major effect via IL-17A, Th1 could play a role through the TNF superfamily.

Soluble TNF like weak inducer of apoptosis is a member of the TNF superfamily, which can be associated with the pathogenesis of celiac disease. In many previous studies, it was thought that TWEAK could play a part in the pathogenesis of autoimmune/chronic inflammatory diseases.^[21,22]^ Some of these diseases are rheumatoid arthritis, systemic lupus erythematosus, systemic sclerosis, and multiple sclerosis.^[14]^ We have not come across a study that examines TWEAK levels in celiac disease, which is another autoimmune/chronic inflammatory disease.

Our study has found a lower sTWEAK level in celiac patients than in the control group. The sTWEAK level was also found to be lower in patients who did not adhere to the gluten diet, and who were autoantibody-positive than in patients who complied with the diet and who were antibody-negative. Furthermore, a negative correlation was determined among the AGA-IgA and the Anti-t TGA levels and the sTWEAK level. Previous studies have shown that inflammation is higher in patients who do not adhere to the diet and have high levels of autoantibodies.^[17]^ However, our study has determined a higher CRP level, which is an indicator of chronic inflammation, in celiac patients than in the control group. There are two ways we can explain how, in our study, the sTWEAK level, a proinflammatory cytokine, is lower in the control group in this high inflammatory disease. First, this could be due to the free form of the sTWEAK level, which increases in the case of inflammation, being determined to be low after binding with Fn14 receptors.^[23]^ Second, it could be due to the increase of the scavenger receptor CD 163 level, which causes sTWEAK to be decayed by inflammatory macrophages in the case of inflammation.^[24]^ A negative correlation being determined between CRP and the sTWEAK level in our study confirms the hypothesis that sTWEAK could decrease with inflammation. The association determined with autoantibodies shows that sTWEAK could have an effect on the increasing autoimmune response.

In our study, the IL-17A level was determined to be higher in celiac patients than in the control group. The IL-17A level was determined to be higher in patients who did not adhere to the gluten diet and who were autoantibody-positive than in patients who complied with the diet and who were antibody-negative. Furthermore, a positive correlation was determined between CRP and celiac antibodies and IL-17A. All of these results show that the increasing IL-17A level could be associated with the pathogenesis of celiac disease, which is an autoimmune/chronic disease. Previous studies that have examined the association between celiac patients and IL-17A have found a high level of IL-17A in celiac patients feeding on gluten, patients with villous atrophies in their mucosal biopsies and patients with positive autoantibody serology.^[5,25–27]^ If we evaluate the results of our study along with previous studies, we can assume that IL-17A could play a part in the inflammatory process in celiac disease, could be correlated with disease activity and associated with celiac autoimmunity.

A negative correlation was determined between sTWEAK and IL-17A and CRP. We can attribute this to the increase of IL-17A along with the increase of inflammation, and the decrease of the free form of sTWEAK, which is another increasing proinflammatory cytokine, by binding to the Fn14 receptor.^[23]^

The main limitation of our study is that our study is cross-sectional. Another limitation is the absence of a study on the Fn14 and CD 163 receptor levels along with sTWEAK levels in celiac patients. If the Fn14 and CD 163 receptors had been studied, we could determine more clearly the actual reason why the sTWEAK level in celiac patients was lower. Because, as we stated before, after acute inflammation starts, scavenger receptors and fn14 receptors rapidly increase and start to link to sTWEAK. Therefore, sTWEAK levels in serum start to decrease.

Lastly, another limitation is the celiac antibody levels in the control group not being studied. If we could attain data on the autoantibody levels of the control group, we could determine whether sTWEAK and IL-17A are independent risk factors for celiac disease. Because, in order to understand the presence of cytokines such as IL-17-A/sTWEAK in the inflammation pathogenesis of the celiac disease, different levels of cytokines, other than the control group, and their determinations as predictors of the disease are precious.

## 5 Conclusions

Consequently, sTWEAK and IL-17A levels were found to be different from the control group for celiac disease. For the subgroups consisting of celiac patients, the levels of these cytokines were coherent with gluten-free diet and different according to the autoantibody positivity. These conditions signify that sTWEAK and IL-17A may be correlated to the inflammation in celiac pathogenesis. In order to clearly understand the importance of sTWEAK and IL 17A in celiac pathogenesis, it is required to administer the anti-sTWEAK and anti-IL17-A treatments on active celiac patients and follow the clinical results.
